# Children’s Spatial Representations: 3- and 4-Year-Olds are Affected by Irrelevant Peripheral References

**DOI:** 10.3389/fpsyg.2015.01677

**Published:** 2015-11-10

**Authors:** Markus Krüger, Georg Jahn

**Affiliations:** ^1^Entwicklungspsychologie und Pädagogische Psychologie, Institut für Psychologie, Ernst-Moritz-Arndt-Universität GreifswaldGreifswald, Germany; ^2^Institute for Multimedia and Interactive Systems, University of LübeckLübeck, Germany

**Keywords:** spatial cognition, spatial orientation, spatial updating, spatial representation

## Abstract

Children as young as 3 years can remember an object’s location within an arrangement and can retrieve it from a novel viewpoint (Nardini et al., 2006). However, this ability is impaired if the arrangement is rotated to compensate for the novel viewpoint, or, if the arrangement is rotated and children stand still. There are two dominant explanations for this phenomenon: self-motion induces an automatic spatial updating process which is beneficial if children move around the arrangement, but misleading if the children’s movement is matched by the arrangement and not activated if children stand still and only the arrangement is moved (see spatial updating; Simons and Wang, 1998). Another explanation concerns reference frames: spatial representations might depend on peripheral spatial relations concerning the surrounding room instead on proximal relations within the arrangement, even if these proximal relations are sufficient or more informative. To evaluate these possibilities, we rotated children (*N* = 120) aged between 3 and 6 years with an occluded arrangement. When the arrangement was in misalignment to the surrounding room, 3- and 4-year-olds’ spatial memory was impaired and 5-year-olds’ was lightly impaired suggesting that they relied on peripheral references of the surrounding room for retrieval. In contrast, 6-years-olds’ spatial representation seemed robust against misalignment indicating a successful integration of spatial representations.

## Introduction

Imagine sitting at a desk with several indistinguishable cups turned upside down. A colleague slips a glass bead under one of these cups. You get up, walk around the desk and sit down at the desk again. Of course, you can retrieve the bead. You have remembered the bead’s location with respect to your own body and with respect to the array of cups and the boundaries of the desk as local landmarks. In principle, you could have remembered the object’s location with respect to boundaries of the room or distal landmarks such as the door, windows, or pictures on the walls. These are all assets even very young children can use for orientation: 16-month-olds can retrieve object locations using dead reckoning or inertial navigation (cf. Newcombe, 1988; Newcombe et al., 1998). And at the age of 21 months or older children successfully include landmarks in egocentric, body-relative representations (Newcombe et al., 1998).

Now imagine again sitting at your desk while your colleague hides the bead. This time, however, your colleague turns the desk by 135° within the room to confuse you. Although the distal landmarks and room boundaries have shifted with respect to the hidden bead, you will be able to retrieve the bead. Interestingly, young children may be impaired by this relative shift of distal landmarks and room boundaries in such a place learning task.

**FIGURE 1:**
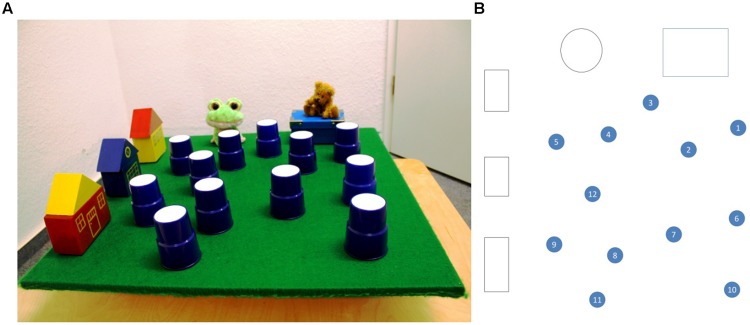
**(A)** Is a photo of the array seen from the children’s perspective. **(B)** Is a schematic overview. The array consisted of a wooden board (70 cm × 70 cm) covered with a green piece of cloth. On the left side of the board a row of three toy houses was placed and on the far side from the children’s point of view a toy frog and an on a box sitting teddy bear were placed. All these objects were placed 2.5 cm off the rim. The houses were 5.5 cm in depth and the toy animals were 11 cm in depth. This left an area 62 cm wide and 56.5 cm deep for the hiding spots. The exact coordinates of the hiding spots can be found in Supplementary Table S1.

In a previous study Nardini et al. (2006) used a landmark shift task similar to the introductory example to study the development of the ability to rely on local landmarks only. In their study landmark shifts were produced in such a way that participants needed to suppress egocentric coding in addition to devaluing distal information. Nardini et al. (2006) asked children from 3 to 6 years to retrieve a toy that was hidden under one of several cups which were arranged in an irregular array on a board (similar to the array in **Figure 1A**). The study comprised two conditions without landmark shifts (*neither move, child move*) and two conditions with landmark shifts (*both move, array move*): either the array and participant stayed (neither-move) or participants walked around the array about 135° (child-move); and children walked along as the array was rotated about 135° (both-move) or solely the array was rotated about 135° (array-move). During these changes the array was hidden from view.

Children of all age groups were most successful when the array stayed in alignment with the room (neither-move, child-move). This was especially true for the youngest age group: the 3-year-olds’ search performance was not even above chance when the alignment changed (both-move, array-move). However, when the alignment changed children were slightly better when their perspective on the array remained the same (both-move).

Nardini et al. (2006) concluded that participants did not solely rely on their perspective on the array, but also encoded hiding places in relation to the surrounding room. This might be seen as an indication for an allocentric representation even in 3-year-olds (cf. Piaget and Inhelder, 1967). An additional factor to bear in mind is the updating of spatial representations by vestibular, proprioceptive, and optic flow information in the course of self-movement (cf. Simons and Wang, 1998; Wang and Simons, 1999; Nardini et al., 2006; Wolbers et al., 2008; Jahn et al., 2012).

This process is called *spatial updating* by Simons and Wang (1998; Wang and Simons, 1999). In their experiments adults were presented with an array of different objects. This array was then hidden from view and the position of one of the objects was changed. Then participants either had to walk a specified path around the array or the array was rotated correspondingly or both. Accuracy in identifying the shifted object was better when participants changed their position. The accuracy decreased when the array was rotated, and when participants moved *and* the array was rotated as well. Simons and Wang (1998; Wang and Simons, 1999) assumed an automatic updating process initiated by self-movement. This process supports spatial orientation – hence participants’ advantage when they changed position. Their orientation becomes maladjusted when the spatial-updating process is triggered although the target moves parallel to the subject. Therewith spatial updating is viewpoint-independent and egocentric (in contrast to allocentric), because the subject remains the center of the representations.

The study by Nardini et al. (2006) was not designed to differentiate between effects of spatial updating and effects of the surrounding room. They point out that according to Burgess et al. (2004) at least in adults both factors contribute to the observed results. It could be argued from a theoretical point of view, that spatial updating alone suffices to explain these results: when children walk around the array, spatial updating takes place. This is adaptive when the array stays still (child-move) and maladaptive when the array moves with the children (both-move). When the array is rotated the egocentric representation is broken (array-move) and when everything stays the egocentric representation remains intact (neither-move). Encoding the surrounding room is not necessary in this approach.

Therefore, our present reference shift experiment was designed (1) to isolate a possible influence of the surrounding room^1^ on young children’s object retrieval performance and (2) to test for effects of spatial updating by self-motion:

(1) So, the viewpoints on the array at encoding and at retrieval match in all conditions of the present experiment. But the array’s alignment with the surrounding room either differs from encoding at retrieval or is shifted. If participants unnecessarily include information concerning the surrounding room for retrieval in this task, performance should drop if the array is in misalignment.
(2) In one condition we rotated the children together with the array on a platform within the room while in the other condition the children walked along the platform. In the latter case self-motion as a cause for spatial updating of the body-relative location representation is avoided, while other possible sources (e.g., optical flow) that might trigger spatial updating remain intact. On the one hand, this might enhance performance, when the array stays in alignment with the surrounding room, as self-movement supplies additional control and information concerning orientation. It might decrease when the array is rotated out of alignment with the surrounding room, because the spatial updating process becomes dysfunctional in this case. This should result in an interaction between an effect of alignment and an effect of self-motion. On the other hand, self-movement might generally decrease performance, as it constitutes an additional task that might strain resources needed for the retrieval task.

## Materials and Methods

### Participants

In total 120 children participated in this study. They were equally distributed among four age groups: there were 30 three-year-olds (mean age = 3 years 4 months, *SD* = 4 months; 14 boys, 16 girls), 30 four-year-olds (mean age = 4 years 4 months, *SD* = 3 months; 18 boys, 12 girls), 30 five-year-olds (mean age = 5 years 4 months, *SD* = 3 months; 20 boys, 10 girls), and 30 six-year-olds (mean age = 6 years 5 months, *SD* = 3 months; 14 boys, 16 girls).

All children were tested in the same laboratory room at our research center. They participated on a voluntary basis and with the consent of their parents.^2^ They were rewarded with a toy after test completion. Parents were reimbursed for their expenses. During the test accompanying parents waited in our lounge.

None of the participants was aware of the purpose of our study or had partaken in a similar study before. All participants were recruited from families registered in Mecklenburg-Vorpommern, Germany.

### Materials

An array closely resembling the one used by Nardini et al. (2006) was constructed (see **Figures 1A,B** for details): on two connected sides of a board a number of child-orientated objects were placed for spatial reference. Twelve up-side-down cups were distributed on the board as potential hiding spots. The array was put on a vehicle. This vehicle consisted of a 200 cm long and 100 cm wide platform on four adjustable wheels. The array was firmly fixed on the frontal end of the vehicle’s platform with the array’s surface 11 cm above it. The spot on which children were supposed to sit down after having mounted the vehicle was marked with a cushion. At the back of the vehicle a handle was attached to allow for the experimenter to maneuver the vehicle. The array could be completely shielded from view with a wooden cover.

All the experiments took place in the same 22.5 m^2^ L-shaped room. A 4.1 m × 3.7 m rectangular area was set apart for the experiment while all the furniture was stored in a 2.2 m × 2.8 m wide expanse. There were a door and a curtained off window in two adjoining walls bordering the experimental area.

### Procedure

The experiment was introduced to the children as a hide-and-seek game. They were asked to mount the vehicle. While being watched by the children the experimenter hid an item under one of the cups. Children were asked whether they had seen where the toy was hidden. The array was then concealed with the wooden cover. The children were told that in the actual game the experimenter would turn the vehicle or ask them to walk around the vehicle or both (see the four different experimental conditions below). Hereafter the cover was lifted and it was the children’s turn to point out the hiding place with a pointer stick. Furthermore children were instructed not to mount, dismount, or walk without being told by the experimenter.

After this short introduction – when children had found the toy and indicated that they had understood the procedure – the actual test started. There were four different experimental conditions that were tested in a within-subjects design. In all these conditions children sat on the vehicle and the experimenter hid a toy under one of the cups, asked the children whether they had seen where the toy was hidden, covered the array, uncovered the array, and asked the children to indicate the position of the hidden toy. The indicated cup was lifted by the experimenter and if this cup did not reveal the hidden toy the experimenter lifted the right one. However, between the covering and uncovering of the array children either (a) remained seated and the vehicle was turned about 360° (*Room+ Child Sits Condition)*, (b) dismounted and were walked around the vehicle and remounted (*Room+ Child Walks Condition)*, (c) remained seated and the vehicle was turned about 135° (*Room– Child Sits Condition)*, or (d) dismounted, the vehicle was turned about 135° while the children watched, and then the children were walked along the corresponding path and remounted (*Room– Child Walks Condition)*. The time needed for self-movement was slightly larger than riding the vehicle, because children had to mount and dismount. Also the time needed for the 135° conditions was slightly shorter than the time needed for the 360° conditions. In all four conditions, the children’s view on the array was the same when the toy was hidden and when children were asked to find the toy. Only in the conditions *Room– Child Sits* and *Room– Child Walks* the relation between the surrounding room and the array was disrupted.

Each condition was realized in four trials; this led to 16 trials in total. Trials were presented in a quasi randomized order. Thirty sets of the 16 trials were compiled with the goal (a) to use as many different targets for every individual set as possible without any target being used consecutively and (b) to use each target evenly over all sets. Each of these 30 sets was used once in every age group.

## Results

To quantify search performance, a performance score was computed for every trial (cf. Nardini et al., 2006) relating the observed error distance between the center of the target cup and the center of the chosen cup to the chance distance in the respective trial. The chance distance was computed as the mean of the distances between the center of the chosen cup and the centers of all twelve cups including the target cup. The performance score was computed as 100*(chance distance – error distance)/chance distance. Thus, a score around zero can be interpreted as performance at a chance level (i.e., participants are guessing).

**FIGURE 2:**
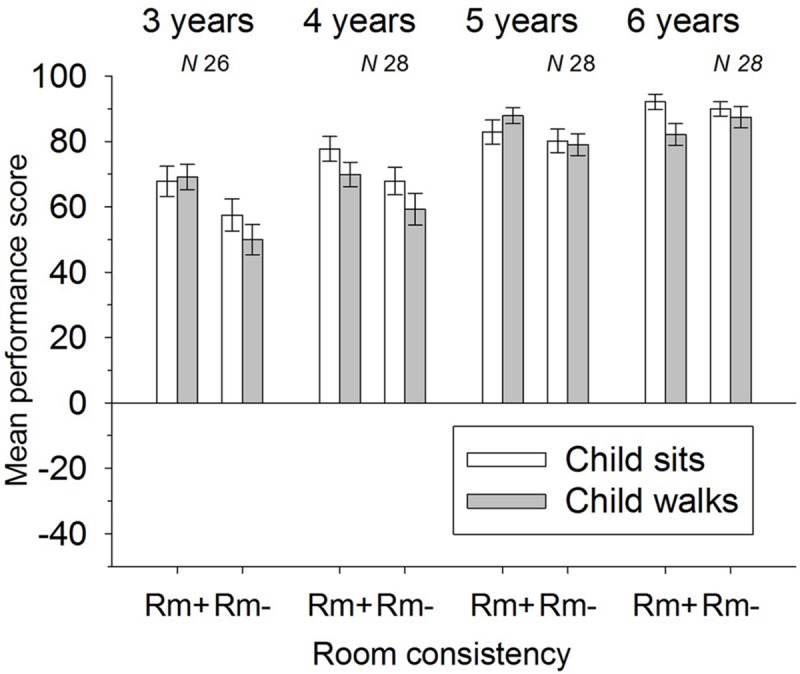
**Mean performance scores and standard errors of the different age groups and experimental conditions.** Room consistency is given as Room+ for array’s ending position consistent with room and Room– for array inconsistent with room (135° rotation).

For the final analysis 10 children had to be excluded: two 6-year-olds, because they received the wrong test; two 3-year-olds, because the experiment had to be aborted due to non-compliance; one 3-year-old, because the data set was lost due to a technical error; and one 3-year-old, two 4-year-olds, and two 5-year-olds, because their consistent negative performance score implied that they were just guessing or choosing wrong targets on purpose. Of the remaining 110 children, 26 were 3-year-olds (mean age = 36 years 4 months, *SD* = 4 months), 28 were 4-year-olds (mean age = 4 years 5 months, *SD* = 3 months), 28 were 5-year-olds (mean age = 5 years 4 months, *SD* = 3 months), and 28 were 6-year-olds (mean age = 6 years 5 months, *SD* = 3 months). An overview of the remaining children’s performance scores can be found in **Figure 2**.

An ANOVA including the within-subjects variables Room (*Room+, Room-*), Movement (*Child Sits, Child Walks*), and the between-subjects variable Age-group (3, 4, 5, and 6) were computed. There was a significant main effect concerning the factor Room, *F*(1,106) = 20, *p* < 0.001, η^2^ = 0.16, qualified by a significant interaction for the factor Room and Age-group, *F*(3,106) = 4.48, *p* = 0.005, η^2^ = 0.11. Furthermore, there was a significant main effect for the factor Movement, *F*(1,106) = 5.49, *p* = 0.021, η^2^ = 0.05, indicating that children were generally more precise when they remained seated in front of the display (*M* = 77.3, *SD* = 18.1) than when they were made to move around (*M* = 73.3, *SD* = 18.6). No further significant or marginally significant main effects or interactions were found.

To resolve the interaction between Room and Age-group, four additional ANOVAs for the separate age-groups were computed. There was a significant main effect concerning the factor Room for the 3-year-olds, *F*(1,25) = 13.76, *p* = 0.001, η^2^ = 0.36, for the 4-year-olds, *F*(1,27) = 7.08, *p* = 0.013, η^2^ = 0.21, and a marginally significant effect for the 5-year-olds, *F*(1,27) = 3.98, *p* = 0.059, η^2^ = 0.13, but no such effect was discernable in the 6-year-olds, *F* < 1, *p* = 0.45. Indeed, children were more precise when the display’s ending position was consistent with the room (3-year-olds: *M* = 68.5, *SD* = 15.8; 4-year-olds: *M* = 73.8, *SD* = 14.0; and 5-year-olds: *M* = 85.4, *SD* = 13.2) than when the display’s ending position was inconsistent with the room (3-year-olds: *M* = 53.8, *SD* = 19.3; 4-year-olds: *M* = 63.6, *SD* = 20.0; and 5-year-olds: *M* = 79.6, *SD* = 13.3).^3^

**FIGURE 3:**
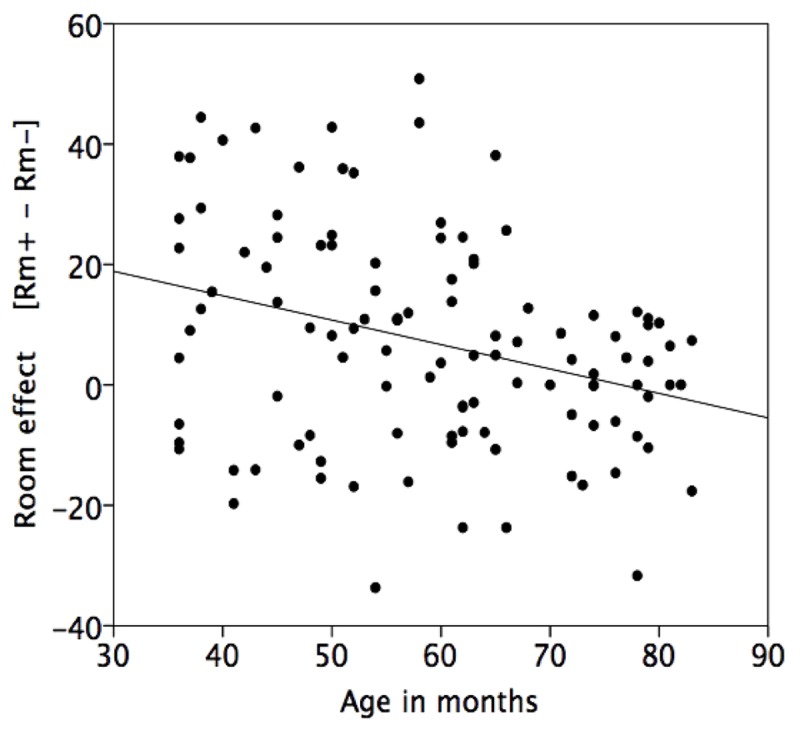
**Regression of the difference score reflecting the effect of the landmark shift (difference between the mean performance score in the Room+ and the Room– conditions) on age in months**.

An additional regression involving age in months and the difference between Room+ and Room– confirmed the age trend concerning the Room effect, standardized coefficient *b*^∗^ = –0.32, *t*(108) = –3.47, *p* = 0.001, adjusted *R*^2^ = 0.09 (see **Figure 3**). No reliable difference between the performances of boys and girls was found, *p* > 0.10.

## Discussion

The better retrieval performance when the display was consistent with the room clearly confirms the assumption that young children tend to encode the targets including distal references of the surrounding room, even when their perspective on the array is kept stable (cf. Nardini et al., 2006).^4^ At the same time the manifest age trend shows children’s advancement to more successful retrieval (see **Figure 3**). Performance is not only getting better with age overall, but the effect of room consistency that is evident in the 3-year-olds and 4-year-olds, is barely detectable in the 5-year-olds, and vanished in the 6-year-olds.

The drop in performance when children walked along the vehicle – especially concerning the 4- and 6-year-olds – might be attributed to an automated change in spatial representation that is maladaptive as the perspective on the array is kept stable, because participants’ movement is matched by the rotation of the array. However, according to this interpretation one would expect an interaction between the room effect and the movement effect because no such performance loss should occur, when the array is rotated about 360° and the children’s movement matches this rotation. Then again, Simons and Wang (1998; Wang and Simons, 1999) make no prediction about what happens, when spatial updating should cancel itself out. Therefore, the apparent difficulties resulting from walking around the vehicle might simply be due to the movement accidentally interfering with children’s focus on the task.

Still, this does not answer the question, why there is no interaction discernible between room and movement. One explanation might be that spatial updating is not dependent on active self-movement. At least in adults spatial updating can be triggered by passive movement: Wang and Simons (1999) moved adults seated on an office chair around an array and found the same effect on object identification as described in the Introduction. On the one hand – in hindsight – it does not seem unreasonable to expect that optical flow or the vestibular system is sufficient to detect movement and elicit spatial updating in children, too. Obviously, controlling only one of at least three possible factors triggering spatial updating was not enough. On the other hand, the fact that the vehicle and the array constituted one unit could have helped children – especially when they rode the vehicle – to regard peripheral references as functionally irrelevant.

This was not the case here. Instead in the younger participants we see the strong tendency to encode peripheral references of the surrounding room and use them for retrieval. This use of peripheral references is generally in line with research concerning children’s reorientation after being disoriented. In principle, all mechanisms currently discussed for reorientation are applicable here: it is possible that room geometry is used by children (Lee and Spelke, 2008, cf. Cheng, 1986; Gallistel, 1990). As our laboratory room has a unique geometrical shape (see Materials) this might have been a salient and stable feature for the younger children. Children of the tested age-groups should have been able to use distal landmarks (Newcombe, 1988; Newcombe et al., 1998) as our room provided doors, windows, and furniture. Even visual snapshots might have played a role in children’s orientation (Piaget and Inhelder, 1967; cf. Diwadkar and McNamara, 1997): when retrieving the object in the Room– conditions the perspective on the array is exactly the same as when the object is hidden. The only change is found in the irrelevant background of the surrounding room. This would amount to younger children paying the same consideration to the relevant center of a visual snapshot as to irrelevant periphery. Anyway, in our experiment no definitive conclusion can be drawn about the exact nature of the distal features used by the younger children and this must be addressed in future experiments by controlling the surrounding room (cf. Burgess et al., 2004).

There is no reason to assume that the older children did not encode peripheral references of the surrounding room or the younger children did not encode the proximal references of the array, but we see a clear trend over age away from erroneously using the surrounding room for retrieval. This empirically clear age trend suggests a more flexible use of spatial information and an underlying shift from the undifferentiated to the meaningful or from random to focused in a cognitive adaptive development (see Nardini et al., 2009; Cheng et al., 2013; cf. Siegler, 1996).

## Conflict of Interest Statement

The authors declare that the research was conducted in the absence of any commercial or financial relationships that could be construed as a potential conflict of interest.

## Footnotes

1 Here, the surrounding room encompasses distal landmarks and room geometry. While there is some discussion which of these is used for orientation, this is not controlled in the present experiment (cf. Cheng, 1986; Newcombe et al., 1998; Burgess et al., 2004).

2 This study was not connected to any form of formal education or medical treatment and approval by the ethics committee was not required. Nevertheless, we can guarantee that we consider possible ethical issues for all our studies. Therefore, the used materials were chosen not to pose any undue risk or harm. All participants gave informed consent or consent was given by their respective parents in written form to partake in this study. Even after consent was given, participation was on a voluntary basis and participants could end their cooperation anytime without any consequences. Proceedings were recorded and participants or their parents were allowed to examine protocols at any time. This proceeding is in accordance with ethical directives specified by the Deutsche Gesellschaft für Psychologie (DGPs). Both authors are members of the DGPs.

3 This additional analysis suggested that the aforementioned main effect for the factor Movement might only be statistically reliable for the 4-year-olds (*p* = 0.026) and the 6-year-olds (*p* = 0.042), but not for the other age-groups (all *p*s > 0.10).

4 Although effects of the minimal handling time differences between the 135° trials (Room–) and the 360° trials (Room+) cannot be ruled out, it must be noted that the Room– trials had the shorter duration and therefore should have given participants – if anything – an advantage.
