# Supplementary material for: Children’s Spatial Representations: 3- and 4-Year-Olds are Affected by Irrelevant Peripheral References
Source: Front Psychol. 2015 Nov 10;6:1677. doi: 10.3389/fpsyg.2015.01677 (PMC4639604; doi:10.3389/fpsyg.2015.01677)
Supplement: Supplementary file 1 [file Table_1.PDF]

Supplementary Table

Position of the Cups on the Array

Cup	x in cm	y in cm
1	-11	-23.5
2	-22.4	-28.5
3	-31.1	-17.7
4	-40.3	-25
5	-52.8	-26.7
6	-11.3	-44.3
7	-25.8	-48
8	-39.3	-52.8
9	-53.3	-50.3
10	-12.4	-60.7
11	-43.4	-63
12	-44.5	-38.7

Note. All distances are measured from the upper right corner of the array as seen by the children.

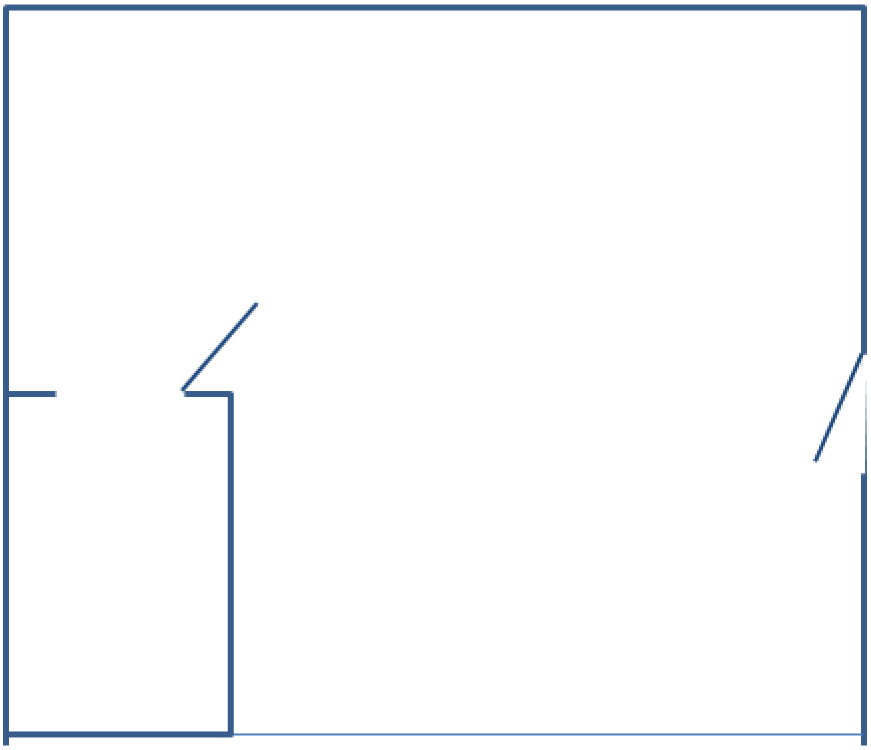

Supplementary Figure. Ground plan of the laboratory room.
